# An electrochemical sensor based on a modified glassy carbon electrode for detection of epinephrine in the presence of theophylline

**DOI:** 10.5599/admet.2082

**Published:** 2024-04-15

**Authors:** Zahra Sarbandian, Hadi Beitollahi

**Affiliations:** 1 Department of Chemistry, Graduate University of Advanced Technology, Kerman, Iran; 2 Environment Department, Institute of Science and High Technology and Environmental Sciences, Graduate University of Advanced Technology, Kerman, Iran

**Keywords:** CeO_2_-ZnO nanocomposite, voltammetry, sensing platform, modified electrode

## Abstract

**Background and purpose:**

Neurotransmitters are chemical messengers that enhance and balance signals between cells and target cells in the body. They are vital to the body's ability to function. Epinephrine is one of the most essential catecholamine neurotransmitters with an important biological and pharmacological role in the mammalian central nervous system. Therefore, it is very important to develop sensitive, simple, and fast methods for the determination of this compound.

**Experimental approach:**

In the present work, a glassy carbon electrode (GCE) modified with the cerium oxide-zinc oxide (CeO_2_-ZnO) nanocomposite (CeO_2_-ZnO/GCE) was developed for the sensitive and quick detection of epinephrine. The CeO_2_-ZnO nanocomposite was prepared by hydrothermal method. Electrochemical methods such as voltammetry and chronoamperometry techniques were used to investigate the performance of the developed sensor.

**Key results:**

The resulting CeO_2_-ZnO/GCE showed a remarkable response towards the determination of epinephrine. The electrochemical sensor demonstrated a wide dynamic linear range from 0.1 to 900.0 μM for analysis of epinephrine. The LOD equalled 0.03 μM for epinephrine. In addition, the electrochemical sensor had good feasibility for concurrent detection of epinephrine and theophylline. Furthermore, experimental outputs indicated that the oxidation peaks of epinephrine and theophylline were separated by a 685 mV difference between the two peaks in PBS at a pH of 7.0. Also, an electrochemical sensor has been employed to analyse epinephrine in real samples (urine and epinephrine Injection).

**Conclusion:**

The good and acceptable analytical performance of the developed sensor can provide a promising tool for the analysis of epinephrine in real samples.

## Introduction

According to the research, epinephrine [1-(3,4-dihydroxyphenyl)-2-methylo-aminoethanol], frequently known as adrenaline, has been introduced as the major catecholamine neuro-transmitters in mammalian central nervous systems (CNS) [1], with the main contribution to transmit nerve impulse. This drug is bio-synthesized in the adrenal medulla and sympathetic nerve terminals and is correlated to diverse physiological processes and diseases [2-4]. In pharmaceutics, epinephrine has extensive use for treating neural dysfunction and in the clinics, it occurs as one of the organic cations in nervous tissues and biological fluids. Epinephrine treats cardiac arrest, asthma, heart block, hypertension, nasal congestion, and so on. It also enhances the heart rate, obstructs the blood vessels, and dilates the airways. Therefore, it results in the “fight or flight response,” energizes diverse biological systems via supplying glucose to the body and contributes centrally to mental or physical stress. Moreover, athletes need epinephrine to improve speed and strength. Such prominent impacts of epinephrine have made it a potential stimulant. For this reason, it is forbidden in athletic competitions by the World Anti-Doping Agency [5-7]. In addition, abnormal level of epinephrine is correlated with several illnesses like cardiac pathologies, Huntington’s, Parkinson’s and schizophrenia [8]. Therefore, detecting and quantifying the above compound would be often useful for neuro-chemistry, medical diagnosis and treatment of the disease.

Theophylline, also known as 1,3-dimethylxanthine, has been proposed as one of the methylated xanthines with relaxation impacts on the smooth muscles of airways in the lungs, which may result in various physiological influences such as relaxing the bronchial muscles, increasing secretion of gastric acid and finally stimulating CNS. This compound has been considered one of the popular medications for chronic asthma [9] and influences the concentration in ranges from 5-20 μg mL^-1^ (55 to 110 μM). Over 20 μg mL^-1^, this drug may cause mild to severe conditions, including fever, arrhythmia, insomnia, dehydration, heartburn, tachycardia, anorexia, coma, as well as respiratory or cardiac arrest [10]. Because of this, it is crucial to have a method that can detect theophylline simply, rapidly, and efficiently.

These compounds (epinephrine and theophylline) are co-existing in the real specimens. So, quick, simplified and inexpensive detection techniques for simultaneous determination of epinephrine and theophylline in biological fluids are of great importance. Several analytical procedures are available for epinephrine and theophylline that include spectrophotometry, high-performance liquid chromatography, capillary electrophoresis, chemiluminescence, surface-enhanced Raman scattering, gas chromatography-mass spectrometry, as well as electrochemical methods [11-22].

Among these techniques, electrochemical methods were preferable and have attracted increasing attention because of their simplified preparation procedure, acceptable sensitivity, quicker responses, very good selectivity, and affordability [23-26]. Some investigators have exploited glassy carbon (GC) in voltammetric experiments. GC is one of the gas-impermeable, electrically conductive materials strongly resistant to chemical attacks [27]. Moreover, electron transfer reactions usually proceed very fast at glassy carbon electrodes (GCE) and thus show faster responses than the thin-film metal electrodes. GCEs are widely used in electroanalysis [28-30].

Recently, researchers have been specifically interested in the modified electrodes because of their very good electrocatalytic activity toward various substances [31-33]. Moreover, nanomaterials are chemical materials fabricated and utilized at a very small scale. They have been devised to exhibit new features in comparison with the materials on a larger scale. Therefore, nanomaterials have attracted increasing interest in different fields [34-36]. Nanomaterials have been highly attractive for constructing sensitive and selective electrochemical sensors due to superior electrical conductivity, acceptable biocompatibility and large surface areas [37-40].

Diverse metal oxide nanoparticles (NPs) are employed to fabricate the electrodes to enhance the function of the electrochemical sensors. Compared with other metal oxide NPs, the CeO_2_ NPs gained remarkable significance due to their specific features like the higher ability for oxygen storage, affordability, innate Ce^3+^/Ce^4+^ redox cyclic, and higher catalytic activities [41,42]. Notably, ZnO NPs enjoy benefits like effective surface modification, narrower size distribution, favourable bio-compatibility, high electron transfer properties and good chemical stability [43,44]. The synergetic effects of composite nanomaterials can differentiate their catalytic performance from each one of the components.

Therefore, in this work, the CeO_2_-ZnO nanocomposite-modified GCE has been employed as the electrochemical sensor for detecting epinephrine. Several electrochemical experiments like cyclic voltammetry (CV), differential pulse voltammetry (DPV) and chronoamperometry (CHA) indicated more acceptable electrochemical function of CeO_2_-ZnO nanocomposite for epinephrine due to higher conductivity as well as higher surface areas of nanocomposite. Also, the modified electrode, CeO_2_-ZnO/GCE, showed separated oxidation peaks for simultaneous analysis of epinephrine and theophylline. Additionally, our sensor displayed high performance for detecting epinephrine in real specimens. The novelty of the presented work is the use of CeO_2_-ZnO nanocomposite-modified GCE as a sensing platform for the first time for the determination of epinephrine in the presence of theophylline.

## Experimental

### Chemicals

All materials with analytical grades applied throughout this work were supplied by Aldrich and Merck. Also, the synthesis and characterization of CeO_2_/ZnO nanocomposite have been reported in our previous work [45].

### Modification of GCE

The modification of CeO_2_/ZnO nanocomposite over the GCE surface was accomplished in this way: 1 mg of CeO_2_/ZnO nanocomposite was suspended in 1 mL of the distilled water for forming the suspension, which was sonicated for 20 min for dispersing the nanocomposite. Finally, a 5 μL aliquot of the suspension was pipetted over the surface of a GCE and drying was done at the ambient temperature.

To calculate the electrochemically active surface area (EASA) of the CeO_2_-ZnO/GCE and unmodified GCE, the cyclic voltammograms (CVs) were recorded at different scan rates in 0.1 M KCl solution containing 1.0 mM K_3_[Fe(CN)_6_] as a redox probe. Using the Randles-Ševčik equation, the value of the EASA for CeO_2_-ZnO/GCE (0.091 cm^2^) was determined to be 2.9 times higher than unmodified GCE.

### Preparation of real samples

The human urine samples were collected and centrifuged for 15 min at 2000 rpm (at ambient temperature). The supernatant was filtered using a 0.45 μm filter and diluted with PBS (pH 7.0). The diluted urine samples were spiked with different amounts of epinephrine. The epinephrine contents were analyzed using the proposed and standard addition methods.

For preparation of epinephrine injection, one mL of injection was diluted with 0.1 M PBS (pH 7.0). Then, different values of the diluted solution were transferred into the volumetric flasks and diluted to the mark with PBS. After that, the epinephrine content was determined by the proposed method using the standard addition method.

### Electrochemical measurements

Electrochemical experiments were recorded using a PGSTAT-302N Autolab potentiostat/galvanostat (Eco Chemie, The Netherlands). The control of all experiments was carried out by a General-purpose electrochemical system (GPES) software. Electrochemical experiments were performed by a three-electrode system containing modified or unmodified GCE as a working electrode, Ag/AgCl (KCl 3 M) as a reference electrode, and platinum wire as a counter (auxiliary) electrode. The cyclic voltammetry (CV) experiments to study the electrochemical behavior of epinephrine at the surface of unmodified and modified GCE were performed in a PBS 0.1 M (pH 7.0) in the potential range *vs.* Ag/AgCl with a scan rate 0.05 V s^-1^. The CV experiments to evaluate the effect of scan rate were conducted in a PBS 0.1 M (pH 7.0) in the potential range *vs.* Ag/AgCl at various scan rates. The chronoamperometric studies were conducted in a 0.1 M PBS (pH 7.0) with a fixed potential of 0.3 V *vs.* Ag/AgCl with a time of 10 s. The differential pulse voltammetry (DPV) measurements were carried out in a 0.1 M PBS (pH 7.0) containing different concentrations of epinephrine with a step potential of 0.01 V and a pulse amplitude of 0.025 V in the potential range.

## Results and discussion

### Electrochemical behavior of epinephrine at the CeO_2_-ZnO/GCE

**Figure 1.**
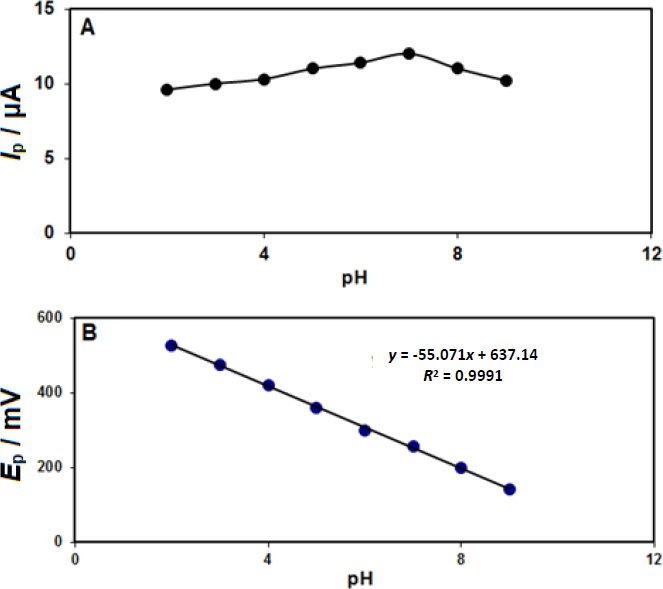
A) Variation of peak currents as a function of solution pH and B) Variation of peak potentials as a function of solution pH obtained from DPVs.

The supporting electrolyte pH significantly affects the epinephrine electrocatalysis on the CeO_2_-ZnO/GCE surface. The pH influence on epinephrine detection in phosphate buffer solution (PBS) on the modified electrode surface was explored at different pH values (2.0 to 9.0) and the epinephrine concentration of 200.0 μM. The maximum peak current of epinephrine oxidation was found at the pH value of about 7.0; accordingly, this value was selected as the optimal experimental condition for the experiments (Figure 1A).

Also, according to the results, epinephrine’s oxidation potential shifted to negative values by increasing pH according to the obtained linear equation (Figure 1B), indicating an equal number of protons involved in the oxidation process.

**Figure 2.**
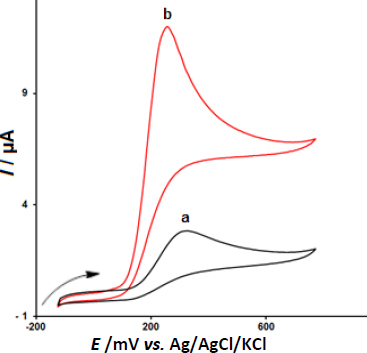
CV responses of 200.0 μM epinephrine at (a) bare GCE and (b) CeO_2_-ZnO/GCE in 0.1 M PBS of pH 7.0.

In the next step, we examined the potential application of CeO_2_-ZnO/GCE for electrooxidation and determination of epinephrine via CV. Figure 2 depicts the CV responses for 200.0 μM epinephrine oxidation on (a) bare GCE, and (b) CeO_2_-ZnO/GCE in 0.1 M PBS of the pH 7.0 at the scan rate 50 mV s^-1^. The oxidation overpotentials decreased (from 330 mV at the bare electrode to 255 mV at the modified electrode) and the peak current increased at CeO_2_-ZnO/GCE compared to the bare GCE.

### Effect of scan rate on the results

**Figure 3.**
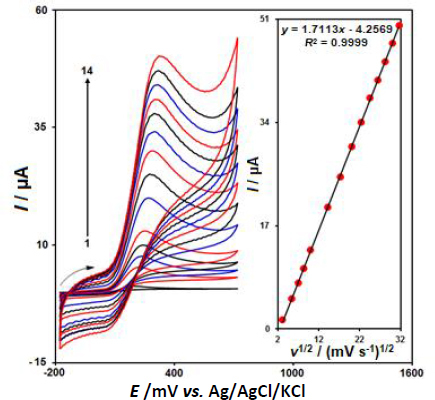
CV curves of 100.0 μM epinephrine in PBS (0.1 M, pH 7.0) at different scan rates (10-1000 mV s^-1^) on CeO_2_-ZnO/GCE (1-14 refers to 10, 30, 50, 70, 100, 200, 300, 400, 500, 600,700, 800, 900 and 1000 mV s^-1^). Inset: Plot of scan rate square root versus epinephrine oxidation peak current.



Figure 3 displayed the CV behaviors of 100.0 μM epinephrine in 0.1 M PBS at the pH value of 7.0 at variable scan rates on the surface of CeO_2_-ZnO/GCE. Based on the results, there was a gradual elevation in the peak current of epinephrine oxidation and a positive shift of the peak potential was observed by increasing the scan rates from 10 to 1000 mV s^-1^. According to Figure 3 (inset), the height of the anodic peak currents of epinephrine fitted to the square root of scan rate (*v*^1/2^), with the regression equation (1):

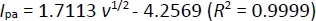

(1)


Hence, this study showed that the electrode reaction was a diffusion-controlled process.

**Figure 4.**
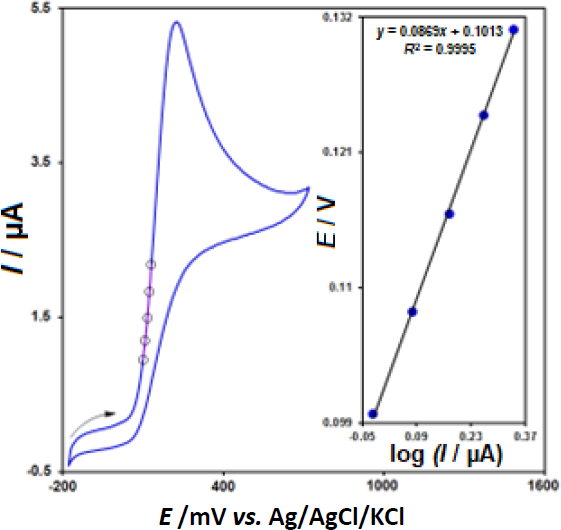
CV response for 100.0 μM epinephrine with 10 mV s^-1^ scan rate and the inset is the Tafel plot derived from the rising part of the corresponding voltammogram.

In order to define the electron transfer coefficient (α) between epinephrine and CeO_2_-ZnO/GCE, we drew Tafel diagram (*E vs.* log *I*) (Figure 4, inset), with the use of an activation area (ascending section) of the voltammogram registered at 10 mV s^-1^ for 100.0 μM epinephrine (Figure 4) and approximated the slope from the linear plot that equaled 0.0869 V^-1^. The slope value equalled *n*_α_ (1- α) *F*/2.3*RT* and, thus, it was possible to estimate α value 0.32 (provided that α = 1).

### Chronoamperometric measurements

**Figure 5.**
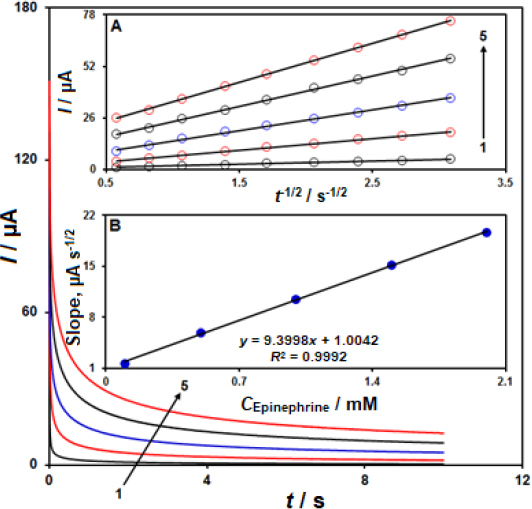
The chronoamperograms obtained on CeO_2_-ZnO/GCE in PBS (0.1 M, pH 7.0) at different epinephrine concentrations; Note: 1-5: 0.1, 0.5, 1.0, 1.5, and 2.0 mM of epinephrine. Inset A - plot of *I* versus *t*^-1/2^ based on chronoamperograms (1-5). Inset B - slope plot of straight line versus epinephrine concentration.

Chronoamperometric records for epinephrine detection on the CeO_2_-ZnO/GCE surface were performed using a working electrode with the potential of 300 mV at variable epinephrine in PBS (0.1 M, pH 7.0), as shown in Figure 5.

The Cottrell equation describes the electrochemical reaction current for epinephrine at the limiting mass transport conditions. Figure 5A shows the experimental plots of *I* versus *t*^−1/2^ with the optimal fits at different epinephrine concentrations. Then, we plotted the slopes of obtained straight lines versus epinephrine concentration, as shown in Figure 5B. Based on the achieved slope and Cottrell equation, the mean *D* value was estimated at 7.45×10^−6^ cm^2^ s^-1^.

### DPV analysis of epinephrine at CeO_2_-ZnO/GCE

**Figure 6.**
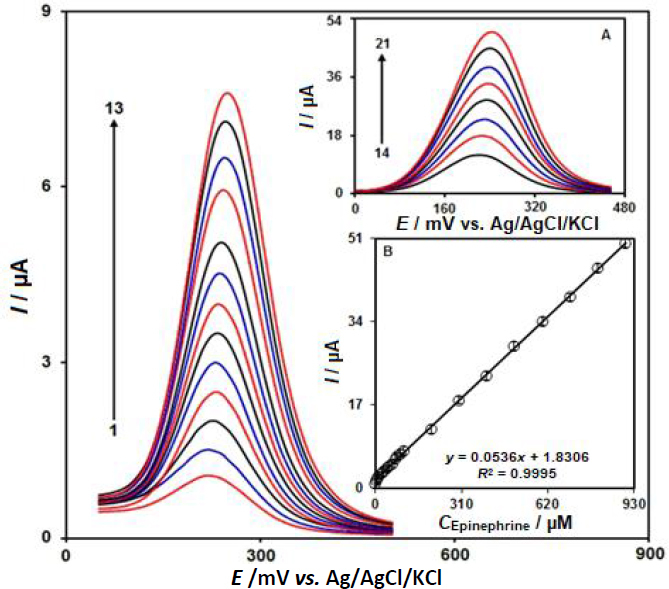
DPV responses of epinephrine at CeO_2_-ZnO/GCE in the concentration range 0.1 μM - 900.0 μM in PBS (0.1 M, pH = 7.0); 1-21 refers to 0.1, 1.0, 5.0, 10.0, 20.0, 30.0, 40.0, 50.0, 60.0, 70.0, 80.0, 90.0, 100.0, 200.0, 300.0, 400.0, 500.0, 600.0, 700.0, 800.0, and 900.0 μM; inset: The calibration curve of DPV peaks against concentration of epinephrine

DPV is a versatile technique for the determination of epinephrine because of the higher sensitivity and lower background current. Differential pulse voltammograms for the determination of epinephrine is shown in Figures 6 and 6A. Figures 6 and 6A show that with increasing concentration of epinephrine from 0.1 to 900.0 μM, the *I*_pa_ increases with a small shift of the oxidation potentials. The plot of *I*_pa_ versus the concentration of epinephrine was plotted as shown in Figure 6B and it shows an almost straight line with good linearity with the linear regression equation *I*_pa_ = 0.0536 *c*_epinephrine_ + 1.8306 (*R*^2^ = 0.9995). The limit of detection (LOD) was calculated as 0.03 μM.

### Simultaneous detection of epinephrine and theophylline at CeO_2_-ZnO/GCE

**Figure 7.**
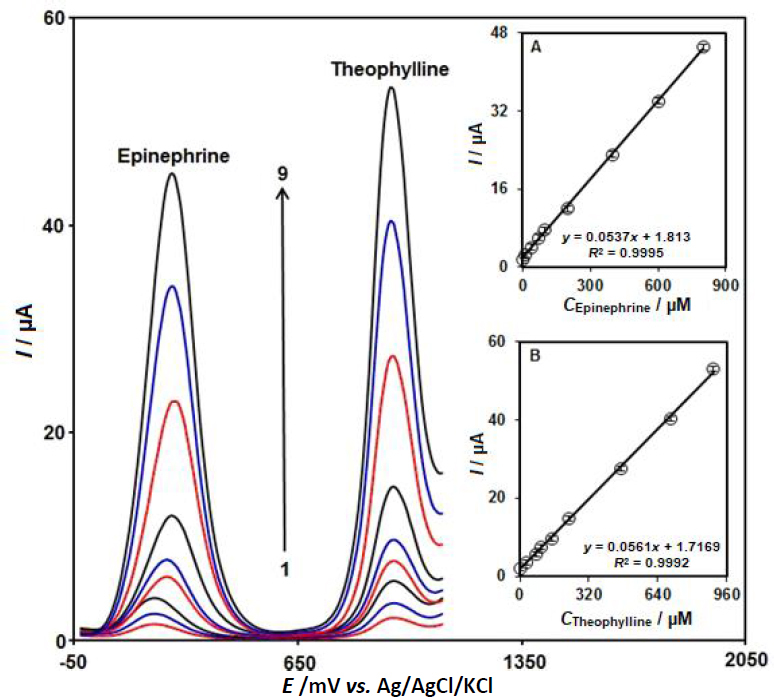
Differential pulse voltammograms of CeO_2_-ZnO/GCE in 0.1 M PBS (pH 7.0) containing different concentrations of epinephrine and theophylline (1-9 refers to mixed solutions of 1.0 + 1.0, 10.0 + 30.0, 40.0 + 75.0, 70.0 + 100.0, 100.0 + 150.0, 200.0 + 225.0, 400.0 + 470.0, 600.0 + 700.0, and 800.0 + 900.0 μM epinephrine and theophylline, respectively). Inset A: Plot of the peak currents as a function of epinephrine concentration. Inset B: Plot of the peak currents as a function of theophylline concentrations

A simultaneous determination of epinephrine and theophylline was carried out in their mixtures by DPV in 0.1 M PBS at a pH of 7.0. Two distinctive oxidation peaks of epinephrine and theophylline can be seen in Figure 7. Moreover, the peak currents for these analytes exhibit a linear increase as the concentration increases without any interference (Figures 7A and 7B). Therefore, a possible simultaneous assay of epinephrine and theophylline could be designed with CeO_2_-ZnO/GCE.

### Stability and reproducibility studies

The stability and reproducibility studies of the CeO_2_-ZnO/GCE sensor for epinephrine determination were evaluated by DPV measurements. The stability of the CeO_2_-ZnO/GCE sensor was tested by storing it at room temperature for several days and used for the determination of 50.0 μM epinephrine in 0.1 M PBS (pH = 7.0) every three days. After 15 days of storage, the peak current was maintained at 94.4% of that of the first day. To evaluate the reproducibility, five CeO_2_-ZnO/GCE were prepared with the same method. The prepared electrodes were used to determine 50.0 μM epinephrine under the same conditions. The RSD of the peak currents was calculated to be 3.4%.

### Sample analysis

To verify the function and possibility of CeO_2_-ZnO/GCE for analyzing the real samples, we detected epinephrine in epinephrine injection and urine electrochemically according to the standard addition approach. Table 1 reports the outputs and achieved recovery percentages of 96.7 to 103.0 % for these samples. These results suggested that CeO_2_-ZnO/GCE exhibits acceptable practical viability for the detection of epinephrine.

**Table 1.** Determining epinephrine in real samples through CeO_2_-ZnO/GCE.

Sample	Concentration, μM		Recovery, %	RSD, %
	Spiked	Found		
Epinephrine Injection	0	-	-	-
	5.0	4.9	98.0	1.7
	10.0	10.3	103.0	2.4
	15.0	15.1	100.7	3.0
	20.0	19.8	99.0	2.5
Urine	0	4.0	-	3.5
	1.0	5.1	102.0	1.9
	2.0	5.8	96.7	2.8
	3.0	7.1	101.4	2.3
	4.0	7.9	98.7	2.0

## Conclusions

In this work, the CeO_2_-ZnO nanocomposite has been synthesized and applied as one of the electrochemical sensors for detecting epinephrine. The CeO_2_-ZnO nanocomposite showed more acceptable catalytic capacities for epinephrine via bimetal oxide synergy. Oxidation peak currents of epinephrine increased proportionally to concentrations between 0.1 and 900.0 μM, and the LOD was 0.03 μM. Also, the CeO_2_-ZnO/GCE exhibited good functions for determining epinephrine in the presence of theophylline with a potential separation of ~685 mV. In addition, the modified electrode showed good stability and reproducibility. The applicability of CeO_2_-ZnO nanocomposite-modified GCE was tested in real samples with good accuracy and satisfying recovery.
